# Enhanced Mechanical Properties of QAl9-4 Aluminum Bronze for High-Speed-Rail Brake Systems with a Pulsed Magnetic Field

**DOI:** 10.3390/ma16175905

**Published:** 2023-08-29

**Authors:** Yujun Hu, Hongjin Zhao, Yinghui Zhang, Bing Zhang, Kefu Hu

**Affiliations:** 1 Faculty of Materials Metallurgy and Chemistry, Jiangxi University of Science and Technology, Ganzhou 341000, China; 2 School of Aeronautical Engineering, Jiangxi Teachers College, Yingtan 335000, China; 3 Guixi Junda Special Copper Materials Co., Ltd., Yingtan 335000, China

**Keywords:** magnetic field treatments, aluminum bronze, microstructure, wear resistance

## Abstract

To improve the mechanical properties and wear resistance of QAl9-4 aluminum bronze alloy parts of high-speed rail brake calipers, the solid aluminum bronze alloy was treated with a pulsed magnetic field in which the magnetic induction intensity was 3T at room temperature. After that, a tensile test and a friction and wear test were carried out on the alloy. The results indicate that the magnetic field promotes the movement of low-angle grain boundaries less than 2° and splices to form subcrystals or fine crystals, which reduces the mean grain size of the alloy. The disordered dislocation changed into a locally ordered dislocation line, the dislocation distribution became uniform, and the dislocation density increased, which simultaneously improved the alloy’s tensile strength and elongation. The elongation increased by 10.2% compared with that without the magnetic field. The increase in strength can provide strong support for the wear-resistant hard phase, and the enhancement of plasticity can increase the alloy’s ability to absorb frictional vibration. Therefore, it was hard for cracks to form and extend, and the specimen’s average friction coefficient was reduced by 22.05%. The grinding crack width and depth decreased, the wear debris became more uniform and fine, and the alloy’s wear resistance increased.

## 1. Introduction

Because of its exceptional qualities, such as high strength, wear resistance, and corrosion resistance, the aluminum bronze alloy is extensively used in high-speed railways, nuclear electricity, ships, and other industries [1]. Tools and equipment now demand more different material qualities as a result of the rapid development of the high-speed railway, nuclear power generation, marine engineering, and other industries. And the aluminum bronze alloy with excellent performance is gradually unable to fulfill the demands of more complex working conditions. Therefore, developing aluminum bronze materials with better performance has recently become a research hotspot. Scholars have carried out a lot of research work to improve the strength, plasticity, wear resistance, corrosion resistance, and other properties of aluminum bronze alloy through electromagnetic stirring, heat treatment, plastic deformation, friction stir treatment, laser treatment, ion implantation, and other technical means and have achieved relatively ideal results [2,3,4,5]. Among them, magnetic field treatment is gradually being paid increasing attention because of its advantages such as non-contact processing, which can enhance the microstructure and properties of metals without changing their shape and appearance. Although establishing a strong magnetic field environment is expensive and difficult in a large space, the significant enhancement in the properties of metallic materials during magnetic-field-assisted heat treatment, especially the enhancement of the properties of workpieces, without affecting their shape and appearance when the magnetic field acts on them alone, has led to rapid and widespread interest.

As an assisted means, magnetic fields can effectively change the strength, plasticity, hardness, and other properties of metallic materials in the process of plastic processing and heat treatment. Refs. [6,7] studied the change in microhardness in aluminum alloys under constant magnetic-field-assisted aging. In comparison, without a magnetic field, the microhardness of the Al-9.25Si-1.5Cu-0.5Fe alloy increased by 25%, whereas that of the Al-5.75Zn-2.3Mg-1.2Cu alloy decreased by 21%. Refs. [8,9] found that compared to the alloy without magnetic-field-assisted aging, the microhardness of the Cu-1.9Be-3.3Ni alloy increased by 38%, whereas the microhardness of the Cu-1.6Be alloy decreased by 25%. Refs. [10,11] reported that after the magnetic-field-assisted annealing of the Ti-6.0Al-4.4V alloy, the elongation of the alloy reached 15.1%, which was 62% higher than that of the alloy without a magnetic field, and the tensile strength was also increased by 2.84%. Ref. [12] investigated the mechanical properties of the cold-rolled 2024 aluminum alloy under a pulse magnetic-field-assisted tensile. The test revealed that the alloy attained excellent mechanical properties, with elongation and tensile strength values of 17% and 410 MPa, respectively. These values were found to be 30.8% and 9.3% higher, respectively, than those of the specimens that were not subjected to a magnetic field.

Furthermore, researchers are increasingly taking magnetic fields as an independent process to study the effect of magnetic fields on the structure and properties of solid metallic materials. Ref. [13] applied a constant magnetic field to the 7055 aluminum alloy alone. The experimental results revealed that the specimen exhibited a tensile strength of 555 MPa, an elongation of 10.5%, and a residual stress of 38 MPa. These values experienced a reduction of 1.8%, an increase of 40%, and a reduction of 68.9%, when compared to specimens that were not treated with magnetic field treatment. In ref. [14], an alternating magnetic field was applied to the cold-rolled NAB bronze alloy and extruded aluminum alloy. In comparison to specimens that were not subjected to magnetic field treatment, the NAB and aluminum alloys exhibited an increase in microhardness of 6.2% and 4.5%, respectively. Additionally, the wear rates of both alloys fell by 61% and 56%, respectively. In the study cited as ref. [15], specimens of EN8 steel and AA2014-T6 aluminum alloy were both put in an alternating magnetic field. The fatigue life of the steel and aluminum alloys were increased by 577% and 605%, respectively, compared with those without magnetic fields. Ref. [16] studied the effect of a pulsed magnetic field on the friction and wear properties of AISI 52100 high-carbon steel. After magnetic field treatment, the friction coefficient of the specimen declined by nearly 13%. Meanwhile, the wear-scar width was reduced by 24%. Ref. [17] presented a study on the impact of pulsed magnetic field treatment on molds made from nickel-based alloys. The results indicated that this treatment brought about a significant improvement in the mean service life of the molds by 34.9%. Additionally, the tensile strength and elongation of the molds were seen to rise by 7.3% and 34.6%, respectively.

The research mentioned above on how magnetic field treatment affects material properties indicates that magnetic field treatment is a useful technique for enhancing the properties of metallic materials. Under the impact of a magnetic field, the properties of ferromagnetic steel and paramagnetic aluminum alloy, magnesium alloy, and titanium alloy are significantly changed. However, their effect on the diamagnetic copper alloy is not significant. The matrix phase of the QAl9-4 aluminum bronze alloy is diamagnetic, and the second phase is paramagnetic. To explore the effect of a magnetic field on the microstructure and properties of the QAl9-4 aluminum bronze alloy, this paper selected an QAl9-4 aluminum bronze alloy rod for a high-speed-railway brake system and directly treated solid alloy samples with a pulsed strong magnetic field at room temperature, with the purpose of investigating the variations in mechanical properties, wear behavior, and microstructure evolution. The research is expected to provide a theoretical and practical reference for expanding the engineering application of magnetic fields, especially for the improvement in the properties of the final formed products with complex shapes.

## 2. Experimental Details

**Figure 1:**
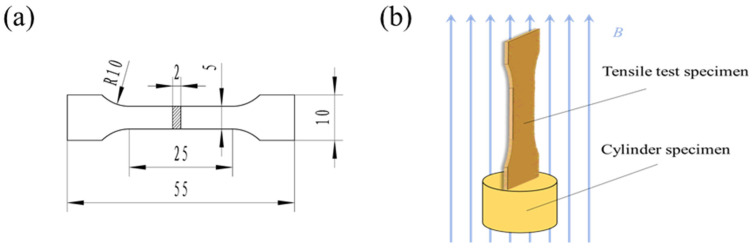
The diagram of magnetic field treated specimen: (**a**) tensile specimen size (unit: mm), (**b**) magnetic-field-treated specimen.

The specimen material used in the experiment was a QAl9-4 aluminum bronze extrusion bar, with a main chemical composition of 8.31 wt.% Al, 2.88 wt.% Fe, and the residual was Cu. The extrusion bar was machined into a Φ13 mm × 15 mm cylindrical specimen and tensile specimen, as shown in Figure 1a, using an electrical discharge machine, and put into the EX-1520-30 pulse equipment for magnetic field treatment. The pulsed equipment output voltage was adjusted to 900 V to control the magnetic induction intensity of 3 T, a pulse interval of 10 s, and a treatment with 30 pulses. The diagram of the magnetic-field-treated specimen is shown in Figure 1b. In this investigation, the effect of thermal effects on the microstructure and properties of the alloy is ignored due to the fact that the maximum temperature observed during the magnetic field treatment does not surpass 50 °C.

**Figure 2:**
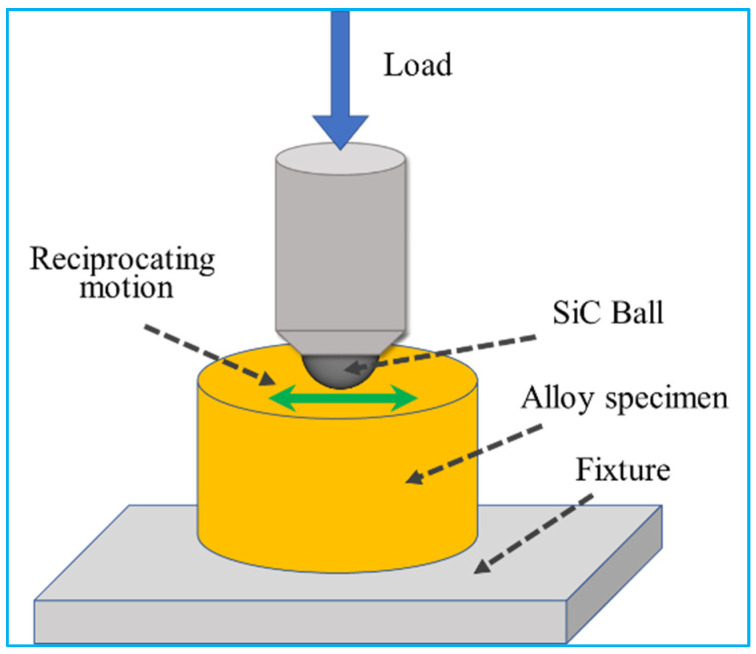
The diagram of reciprocating friction and wear test.

The cross-sections of the specimen that were untreated and treated with a pulsed magnetic field were ground, polished, and etched to prepare metallographic samples for microstructure observation. The sample was etched with a mixture of 5 g FeCl_3_ + 10 mL HCl + 100 mL H_2_O for 20 s. The microstructure of the specimen was observed with a ZEISS SAX10 metallographic microscope, a ZEISS ULTRA 55 emission scanning electron microscope (SEM), and FEI Tecnai G2 F20 transmission electron microscopy (TEM). The grain characteristics and local dislocation density of the specimen were analyzed using a Zeiss-sigma scanning electron microscope HKL technology electron-backscatter diffraction system (EBSD). The phase analysis and residual stress test were performed with an Empyrean X-ray diffractometer (XRD). Tensile testing was performed in accordance with national standards GB/T 34505-2017. The tensile strength and elongation of the specimens were measured with a UTM5105 tensile testing machine at a tensile rate of 2 mm/min. The tensile strength and elongation of each specimen were measured three times and averaged. The microhardness testing was performed in accordance with national standards GB/T 32660.1-2016. The microhardness of the cross-section of the specimen was tested using the 200HVS-5 Vickers hardness tester with a load of 49.8 N and a retention time of 15 s. The microhardness of each specimen was measured at five points, which were averaged. The friction and wear testing was performed in accordance with national standards GB/T 12444-2006. The dry friction and wear test were conducted with the MFT-R4000 high-speed reciprocating friction and wear-testing machine. The experimental parameters were established in the following manner: a vertical force of 10 N was applied, the reciprocating frequency was set at 5 Hz, the reciprocating distance was set at 5 mm, the friction time was set at 30 min, and the friction pair consisted of a Φ3 mm SiC ball. Figure 2 presents the diagram of the reciprocating friction and wear test. The surface topography of the wear marks was quantified using a MicroXAM-3D surface profilometer.

## 3. Results and Discussion

### 3.1. Effect of Magnetic Field Treatment on Dislocations of Alloy

**Figure 3:**
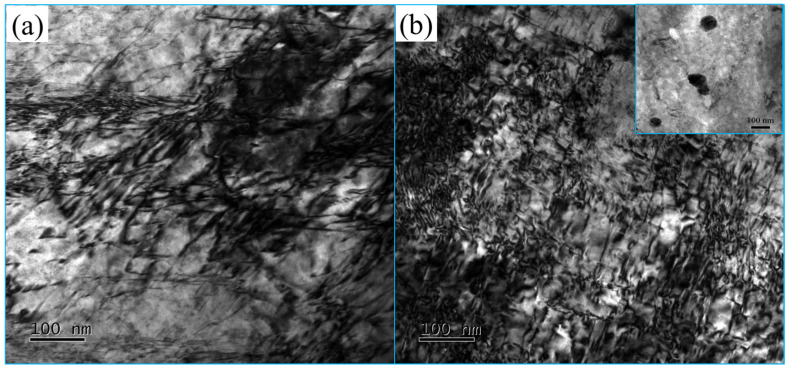
The diagram of the alloy dislocation characteristic: (**a**) untreated, (**b**) treated. The EDS function test of TEM showed that fine AlFe_3_ particles precipitated in the treated specimen and were not found in the untreated specimen. These particles’ morphology is shown in the upper right of (**b**).

Figure 3 shows the dislocation characteristic diagram. The dislocation morphology in the alloy changed obviously after magnetic field treatment. The dislocations in the untreated specimen were basically arranged in disorder and had local dislocation entanglement. The number of dislocations in the treated specimen increased slightly, orderly dislocation lines appeared locally, and the overall distribution of dislocations became more uniform.

In order to conduct a quantitative analysis of the dislocation density, according to the distribution of local misorientation, the kernel average misorientation (KAM) was used to analyze whether the dislocation density is uniformly distributed and judge the degree of plastic deformation or the defect density of the alloy specimen. There exists a positive correlation between the KAM value and the degree of plastic deformation or defect density.

**Figure 4:**
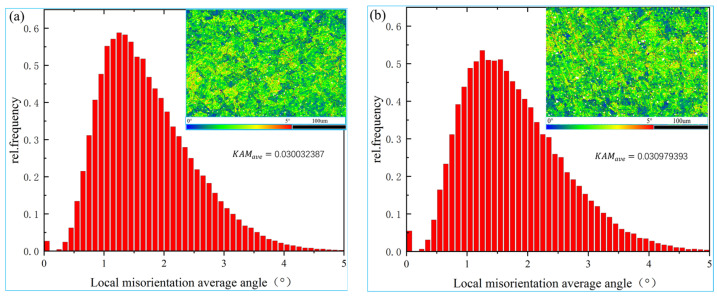
The KAM diagrams: (**a**) untreated, (**b**) treated.

In this paper, the dislocation density changes of specimens that were untreated and treated with the magnetic field were obtained using the KAM diagram. Figure 4 shows the KAM diagram of the alloy specimens. The local orientation difference in the region with a blue color is small, and the local orientation difference in the region with a red color is large. By observing the color distribution and change analysis of the dislocation distribution in each region, it can be found that the yellow and green areas in the KAM diagram of the treated specimen are slightly increased compared with the untreated specimen, and the alloy’s dislocation density has an upward trend.

To more accurately analyze the change in dislocation density, the calculation method employed was in accordance with the strain gradient theory [18,19]:

$$\rho^{GND}=\frac{2{KAM}_{ave}}{\mu b}$$

where $\rho^{GND}$ represents the average geometrically necessary dislocations, *μ* represents the unit length in the EBSD scanning step, *b* represents the magnitude of the Burgers vector, and ${KAM}_{ave}$ represents the average misorientation of the selected area. In this experimental study, *μ* is 0.8 μm, *b* is 0.256 nm, and the ${KAM}_{ave}$ values of the specimens that were untreated and treated with the magnetic field are 0.030032387 and 0.030979393, respectively. In accordance with the above formula, the dislocation densities of the untreated and treated specimens are 2.933 × 10^14^/m^2^ and 3.025 × 10^14^/m^2^, respectively, which again indicates only a slight increase in the dislocation density of the treated specimen.

**Figure 5:**
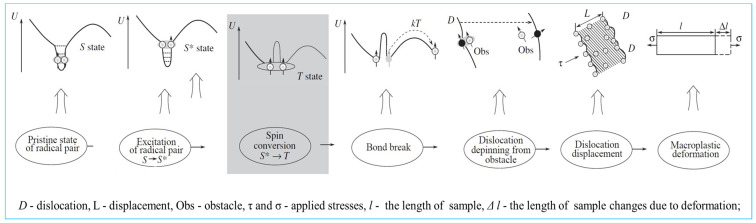
Schematic diagram of magnetic fields speeding up the depinning of dislocations [20].

The increased dislocation density and more uniform distribution indicated that dislocation proliferation and movement occurred. Golovin [20] pointed out that when the distance between the dislocation and the barrier (such as impurity elements and pinning points) is less than nanometers, the interaction between the dislocation and the barrier will occur, thus stimulating free electrons and forming free radical pairs between the dislocation and the barrier. According to quantum theory, pairs of free radicals are divided into a singlet state (S state) and a triplet state (T state). In the S state, the electron spin is counter-parallel, the binding bond is strong, the binding energy is high, and the dislocation is firmly combined with the obstacle. The dislocation needs a higher energy to cross the obstacle. In the T state, the electron spin is parallel, the binding energy of the bond is weak, the binding energy between the dislocation and the barrier is low, and the energy required for the dislocation to overcome the barrier and continue to move is low. At this time, the dislocation can easily cross the barrier. Results of studies have indicated that magnetic fields can promote the transformation of free radical pairs from the S to the T state [20,21]. Figure 5 is the schematic diagram of magnetic fields speeding up the depinning of dislocations [20]. In addition, due to the periodic effect of a pulsed magnetic field on atoms, the diffusion rate of atoms and the interaction between dislocations are accelerated [14,22], and the kinetic barrier for dislocation nucleation is lowered, resulting in dislocation proliferation. In this way, a magnetic field accelerates dislocation depinning, the dislocation movement capacity increases and becomes uniformly distributed, and then the plastic deformation capacity of the material improves.

### 3.2. Effect of Magnetic Field Treatment on the Microstructure of the Alloy

**Figure 6:**
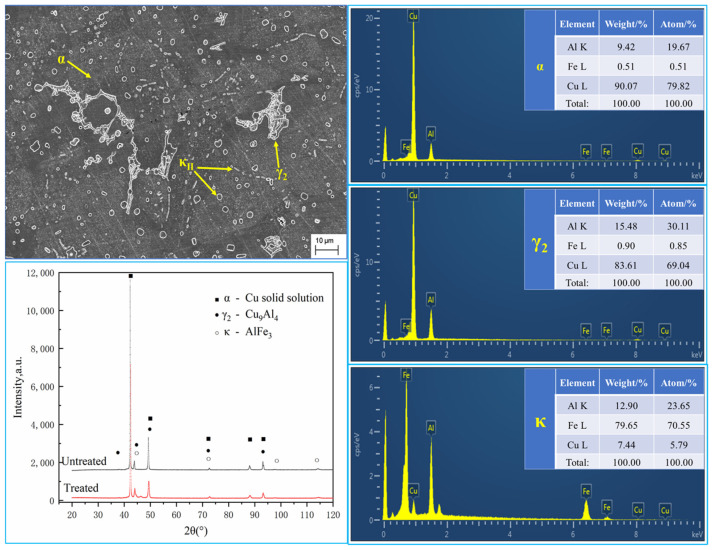
SEM, EDS, and XRD patterns of the alloy specimen.

The microstructure and morphology of the alloy were observed using SEM. It was found that there were three kinds of second phases with different morphologies in the alloy, which were 10–20 μm reticular second phase, 2–5 μm round dot, and a short-rod second phase. To further determine the phase composition of the alloy, an energy spectrum analysis was carried out. Combined with XRD patterns and referring to relevant literature [4,23,24], it can be determined that the reticular second phase of the alloy is the γ_2_ phase (Cu_9_Al_4_ based solid solution), and the round dot and short-rod second phase is the κ phase (AlFe_3_ based solid solution, mainly distributed at the grain boundary or phase boundary), as shown in Figure 6.

**Figure 7:**
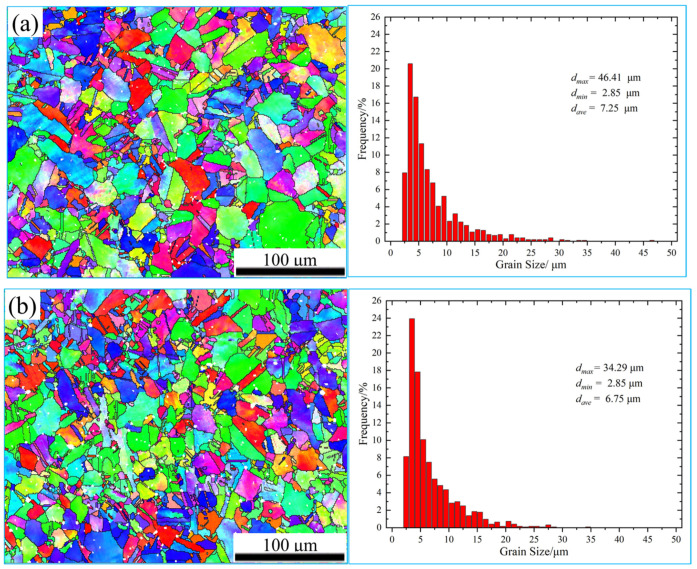
The grain morphology and size distribution: (**a**) untreated, (**b**) treated.

Figure 7 shows the grain morphology and size distribution of the specimen. The grain orientation of the treated and untreated specimens presents an irregular distribution, and the grain-boundary morphology is clear. The average grain diameter of the specimen without a magnetic field treatment was 7.25 μm, and the average grain diameter of the specimen with a magnetic field treatment decreased to 6.75 μm. The proportion of grains with diameters between 3 and 4 μm increased from 20.60% to 23.95% with a magnetic field treatment, indicating that magnetic field treatment can refine the internal grains of the alloy. Further research shows that treated and untreated specimens have standard deviations of their grain size of 5.18 and 4.42, respectively. Magnetic field treatment further reduced grain size differences and made their grain size more uniform.

**Figure 8:**
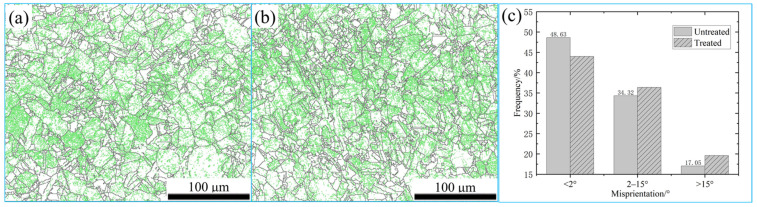
The distribution of grain-boundary misorientation: (**a**) untreated, (**b**) treated, (**c**) grain-boundary content statistics.

The grain-boundary distribution of the specimen can be seen in Figure 8. The green lines indicate low angle grain boundaries (LAGBs) characterized by orientations below 15°, whereas the black lines indicate high angle grain boundaries (HAGBs) characterized by orientations beyond 15°. The proportions of LAGBs in the untreated and treated specimens are 82.95% and 80.38%, respectively. The quantity of LAGBs decreased significantly after magnetic field treatment. Further investigation of the grain-boundary orientation shows that the LAGBs less than 2° decreased obviously from 48.63% to 43.99% after magnetic field treatment. These results indicate that the magnetic field treatment can promote the transition of LAGBs to HAGBs, especially for LAGBs less than 2°.

**Figure 9:**
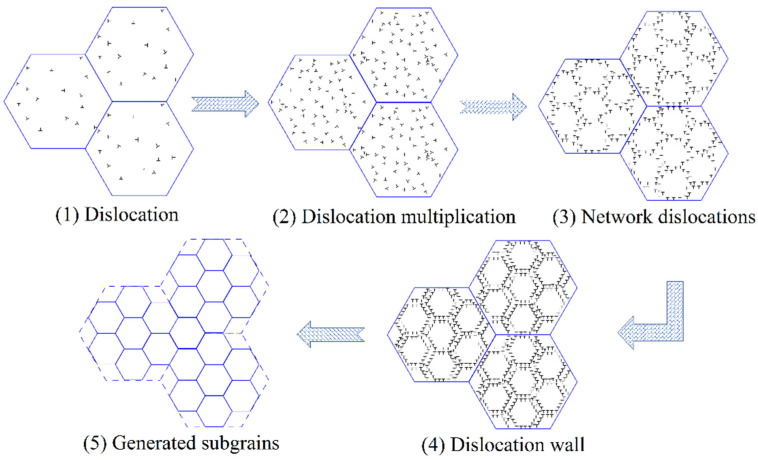
The diagram of magnetic fields promoting grain refinement [28].

After magnetic field treatment, the percentage of LAGBs less than 2° decreased, and the proportion of grains with a diameter between 3 and 4 μm increased most obviously. LAGBs are formed by an arrangement of dislocation arrays, and the development of LAGBs is primarily influenced by the movement of dislocations [25,26]. The application of a magnetic field treatment enhanced the flexibility of dislocation movement, leading to the accumulation of a few dislocations at grain boundaries. The gradual accumulation of dislocations led to the formation of networked dislocations at grain boundaries. This network blocked the migration of other new dislocations, resulting in the accumulation of dislocation pileups. Eventually, the pileups became increasingly reliable and ultimately transformed into a dislocation wall. Through the combined influence of a magnetic field and internal stress, the dislocation acquired sufficient energy to induce the movement of the dislocation wall. Consequently, the dislocation wall merged with another dislocation wall, resulting in the formation of a subgrain. Then, a fine crystal was generated as a result of the interconnections between subgrain boundaries [27]. Therefore, it can be inferred that the reduction in grain size of the alloy is mainly related to the formation of subcrystals or fine crystals via the shifting and splicing of LAGBs under the action of the magnetic field. Figure 9 shows the diagram of magnetic fields promoting grain refinement. 

### 3.3. Effect of Magnetic Field Treatment on Mechanical Properties of Alloy

**Figure 10:**
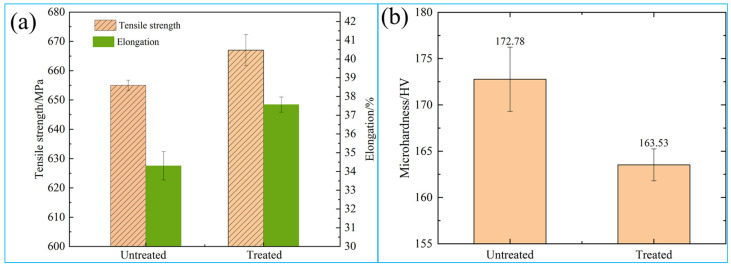
The mechanical properties diagram: (**a**) tensile property, (**b**) microhardness.

Figure 10 shows the mechanical properties of the specimens that were untreated and treated with the pulsed magnetic field. It can be seen from Figure 10a that the tensile strength of the untreated specimen was 653 MPa and the elongation was 34.0%, while the tensile strength of the treated specimen was 663 MPa and the elongation was 37.5%. The tensile strength and elongation of treated specimens each slightly increased and increased by 10.2% compared with untreated specimens. It can be seen in Figure 10b that the microhardness of the untreated specimen is 172.78 HV, and its standard deviation is 3.46 HV. The microhardness of the treated specimen is 163.53 HV, and its standard deviation is 1.72 HV, which decreased by 5.35%.

After magnetic field treatment, the alloy’s strength and plasticity increased simultaneously, while the alloy hardness decreased. That is mainly due to the increased dislocation density and grain refinement under a magnetic field, which improved the alloy strength. At the same time, the dislocation morphology changed from a disorderly arrangement to a locally ordered arrangement. Additionally, the number of dislocation entanglements decreased, and the dislocation distribution became more uniform, which is conducive to improving alloy plasticity. This paper studies the synchronous improvement in strength and plasticity of QAl9-4 aluminum bronze alloy under a magnetic field, and this phenomenon also appeared in Gui. R. L’s [11,12,13] study investigating the mechanical properties of a titanium and aluminum alloy treated with a magnetic field. It can be indicated that magnetic field treatment is a processing method that can realize the synchronous improvement in the strength and plasticity of alloys.

The hardness decline after magnetic treatment is mainly because there is a lot of dislocation tangle and pileup in the alloy without magnetic field treatment. Dislocation movement is promoted after the application of a magnetic field, which causes the dislocation to depin from the barrier and reduces the number of dislocation tangles and pileups. Thus, the stress around the dislocation is decreased, manifested as the change in residual stress at the macro level. The change in residual stress is closely related to hardness. Some studies [29,30] have found that residual compressive stress enhances the hardness of materials, and the existence of residual tensile stress reduces the hardness of the material. The higher the residual compressive stress is, the higher the hardness will be, and the higher the residual tensile stress is, the lower the hardness will be. Therefore, in this paper, an X-ray stress analyzer was used to measure the change in spectral line peak displacement at 93.5° of the alloy (311) crystal plane, and the sin2ψ method [31] was used to measure the residual stresses of the alloy that was untreated and treated with a magnetic field, which were compressive stress and had values of −96.1 MPa and −73.7 MPa, respectively. It can be seen that under the magnetic field, the number of dislocation pileups was less, the distortion degree around the dislocation was reduced, and the residual compressive stress on the alloy surface was reduced. In this way, the hardness of the material was reduced, the dislocation distribution became more uniform, and the hardness distribution was also more uniform.

### 3.4. Effect of Magnetic Field Treatment on Friction and Wear Properties of the Alloy

**Figure 11:**
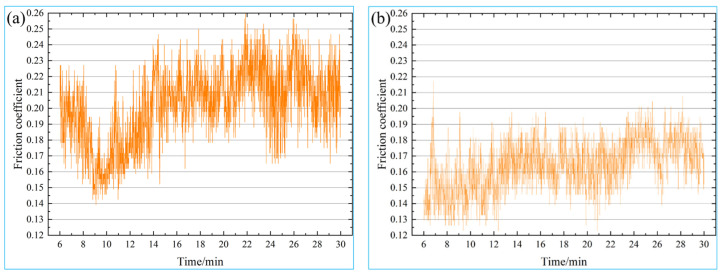
The variation curve of friction coefficient: (**a**) untreated, (**b**) treated.

The friction coefficient is the vital basis to reflect materials’ friction and wear properties. Figure 11 shows the variation curve of the friction coefficient of the specimen. On the whole, the friction coefficient of the untreated and treated specimens fluctuates in a certain range with the change in time, which is mainly caused by reciprocating friction and wear tests and periodic acceleration and deceleration processes for grinding materials. Further analysis shows that the fluctuation range of the friction coefficient of the specimen treated with the magnetic field decreased obviously compared with that without the magnetic field treatment. After analyzing the variation curve of the friction coefficient of the specimen, it can be seen the mean friction coefficient of the untreated specimen is 0.2023, the mean friction coefficient of the treated specimen is reduced to 0.1577, and the mean friction coefficient decreases by 22.05%. By comparing the untreated specimen’s fluctuation amplitude and mean friction coefficient, it can be seen that the treated specimen has a small fluctuation amplitude and a low friction coefficient, indicating that a magnetic field treatment can improve the wear resistance of the aluminum bronze alloy. The friction coefficient is equal to the friction force between the alloy and the friction pair divided by the positive pressure applied to the friction pair. The main reason for the decrease in the friction coefficient of the treated specimen can be attributed to the increase in the hard second phase in the specimen after magnetic field treatment, which effectively supports the grinding material and reduces the contact area between the alloy and the grinding material. As a result, the reduction in the contact area effectively reduces the friction force between the alloy and the grinding material, resulting in a decrease in the friction coefficient.

**Figure 12:**
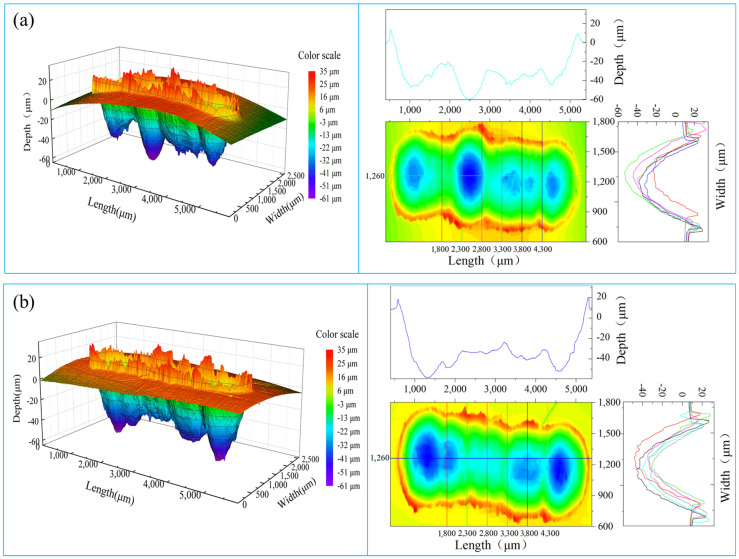
Three-dimensional topography of wear trace profile: (**a**) untreated, (**b**) treated.

The specimen after friction and wear was digitally scanned for the three-dimensional topography of the contour of the wear marks, as shown in Figure 12. The left shows the three-dimensional topography of the wear marks, and the right shows the contour profile of the wear marks. It can be seen from Figure 12 that the middle region of the untreated specimen is deeper, while the two ends are shallower and the overall depth of the wear mark fluctuates wildly. However, the middle region of the treated specimen is shallower, the two ends are deeper, and the fluctuation range of the whole wear depth decreases. The two-dimensional contour data of the width and depth of the wear marks were obtained at 1800, 2300, 2800, 3300, 3800, and 4300 μm in the length direction of the wear marks, and the mean width and depth of the wear marks of the untreated specimen were 671.9 and 390.8 μm, respectively. The mean width and depth of the wear marks on the treated specimen decreased to 661.3 μm and 377.9 μm, respectively, indicating that a magnetic field treatment can improve the wear resistance of the aluminum bronze alloy.

**Figure 13:**
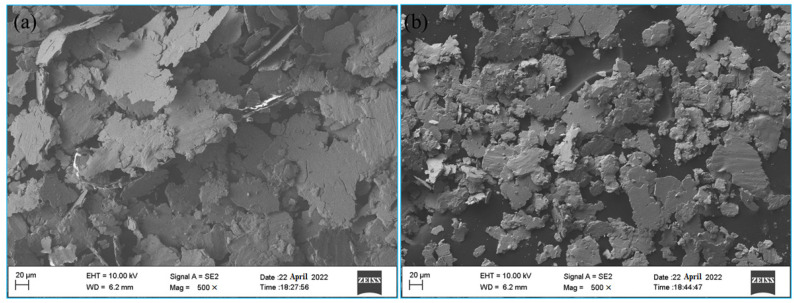
The debris topography: (**a**) untreated, (**b**) treated.

To further analyze the wear behavior of the alloy, the collected wear debris was scanned and observed. Figure 13 shows the SEM morphology of the alloy wear debris. It can be found that the wear debris of the untreated specimen was a relatively larger sheet, indicating that the alloy had more severe wear; the wear debris of the treated specimen was obviously smaller; and the overall size became more uniform. This indicates that the specimen treated with the magnetic field has stronger wear resistance and shows good sliding wear resistance.

**Figure 14:**
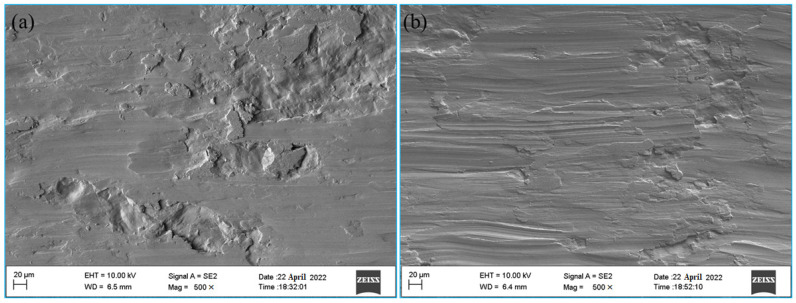
The surface morphology of wear scar: (**a**) untreated, (**b**) treated.

To further verify whether magnetic field treatment can effectively improve the wear resistance of an aluminum bronze alloy, the wear trace morphology was scanned, as shown in Figure 14. The untreated wear surface of the specimen is rough and uneven, and there is a large volume of wear debris sticking on the material surface and a small number of furrows. Under the action of the reciprocating contact stress on the wear surface, the sticky wear debris is turned up again, so the cycle alternates, and then large areas are torn and fall off to form peeling pits and apparent cracks can be seen at the edges of some pits. The wear surface became rough, and the coarser surface aggravated the material wear as the wear continued. At this time, the wear mechanism of the alloy was mainly adhesive wear accompanied by abrasive wear. However, after magnetic field treatment, the peeling pits of the alloy were significantly reduced, the wear surface was smooth, the furrow was shallow and clear, and there was an apparent “plastic flow” trace. In this case, the wear mechanism of the alloy was mainly abrasive wear, accompanied by adhesive wear.

Analysis of the reasons for the formation of wear trace morphology shows that the second-phase particles in the untreated specimen are large and uneven, and there is a large area of matrix phase between the second-phase particles, which have low hardness, so it is easy for adhesion tear and peeling to occur and for large pits to form in the process of wear. After magnetic field treatment, 50–100 nm second-phase particles with high dispersion precipitated inside the specimen, as shown in Figure 3b. The matrix phase and the second phase are evenly distributed, and the area of the matrix phase between the second-phase particles is relatively reduced. In addition, due to the high hardness of the second-phase particles, the deformation and tearing can only be carried out in a smaller area, so the pits formed are small with more furrows.

In conclusion, under the impact of the magnetic field, the alloy’s dislocation density increases and the distribution of dislocation becomes more uniform, the number of the second phase precipitated increases and the distribution is more uniform, and the grain size becomes smaller, which improves the strength and plasticity of the alloy. The improvement in alloy strength provides strong support for the anti-wear hard phase and gives full play to the ability of the anti-wear hard phase to resist wear. With the improvement in the alloy plasticity, the ability to absorb friction and vibration is enhanced [32], it is hard for cracks to form and expand, the alloy has a large capacity for repeated deformation, and it is not easy for peeling to form. These properties enhance the wear resistance of the aluminum bronze alloy.

## 4. Conclusions

The mechanical properties and microstructure of the QAL9-4 aluminum bronze alloy for high-speed-rail brake calipers were changed by the pulsed magnetic field alone, which was mainly reflected as follows.

(1) Under the influence of a magnetic field, dislocation becomes easier to depin and more mobile. At this point, LAGBs less than 2° move and splice to form subgrains or fine grains, thereby reducing the alloy’s average grain size.

(2) After magnetic field treatment, the dislocation density of the aluminum bronze alloy increased, the distribution was more uniform, and the grain size and the residual stress decreased, which led to a synchronous improvement in the tensile strength and elongation of the alloy.

(3) The strength and plasticity of the aluminum bronze alloy were improved by magnetic field treatment, and the nanosecond phase precipitated, which improved the wear resistance of the alloy.

Furthermore, it has been observed that the magnetic field has a certain degree of influence on the properties of the QAl9-4 aluminum bronze alloy. However, this influence is relatively less noticeable when compared to that of paramagnetic alloys. This observation may be related to the fact that the matrix phase of the alloy is diamagnetic and the second phase of AlFe_3_ is paramagnetic. It has been discovered that the presence of a magnetic field leads to an increased precipitation of the AlFe_3_ phase. Hence, it is seen as worth investigating the influence of magnetic fields on the microstructure and properties of aluminum bronze alloys that have a greater amount of second-phase particles.

## Data Availability

Not applicable.
